# Adaptation by Type III CRISPR-Cas Systems: Breakthrough Findings and Open Questions

**DOI:** 10.3389/fmicb.2022.876174

**Published:** 2022-04-14

**Authors:** Xinfu Zhang, Xinmin An

**Affiliations:** ^1^Department of Biochemistry and Molecular Biology, The University of Georgia, Athens, GA, United States; ^2^Beijing Advanced Innovation Center for Tree Breeding by Molecular Design, National Engineering Research Center of Tree breeding and Ecological Remediation, College of Biological Sciences and Technology, Beijing Forestry University, Beijing, China

**Keywords:** CRISPR-Cas system, type III, adaptation, ssDNA secondary structure, reverse transcriptase

## Abstract

CRISPR-Cas systems acquire heritable defense memory against invading nucleic acids through adaptation. Type III CRISPR-Cas systems have unique and intriguing features of defense and are important in method development for Genetics research. We started to understand the common and unique properties of type III CRISPR-Cas adaptation in recent years. This review summarizes our knowledge regarding CRISPR-Cas adaptation with the emphasis on type III systems and discusses open questions for type III adaptation studies.

## Introduction

Prokaryotic cells evolved multiple strategies to defend against viruses and non-beneficial plasmids, including abortive infection, restriction–modification systems (Sturino and Klaenhammer, 2006; Rocha and Bikard, 2022), and recently discovered CRISPR (Clustered Regularly Interspaced Short Palindromic Repeats)-Cas (CRISPR-associated gene) systems (Makarova et al., 2011, 2020b; Hille et al., 2018; Nussenzweig and Marraffini, 2020). The sequence-specific and adaptive defense activity of a CRISPR-Cas system is acquired by adaptation, during which a short fragment (protospacer) of the foreign DNA is captured and integrated into the CRISPR locus at the leader proximal end as a spacer, simultaneously with a duplication of the first repeat (Barrangou et al., 2007; Bhaya et al., 2011; Arslan et al., 2014; Heler et al., 2014). A spacer in a CRISPR array encodes a small CRISPR RNA (crRNA), which guides an interference protein or a protein complex (crRNP) to destroy the previously encountered foreign nucleic acids (Barrangou et al., 2007; Pougach et al., 2010; Sorek et al., 2013). CRISPR-Cas systems are structurally and functionally diverse, and are classified into six types (type I-VI) and multiple subtypes (Haft et al., 2005; Makarova et al., 2006, 2011, 2015; Kunin et al., 2007; Shmakov et al., 2015).

Functional studies of CRISPR-Cas systems, especially those regarding target interference, have inspired researchers to develop many unprecedented, convenient, and powerful tools for genome editing, gene expression control, disease detection and cures, and many other purposes (Pickar-Oliver and Gersbach, 2019). Adaptation abilities of CRISPR-Cas systems and the dynamic CRISPR arrays they generated have been used for bacterial strain typing (Barrangou and Dudley, 2016), bacterial virome detection (Choi and Lee, 2016), and even digital movie encoding and data storage (Shipman et al., 2017). The tremendous contribution of CRISPR-Cas systems to biotechnology makes their fundamental studies invaluable, especially those investigating adaptation, since it is the least understood process of CRISPR-Cas functions. Type III CRISPR-Cas systems have distinct features during target interference (Liu et al., 2018; Molina et al., 2020), and have been repurposed for prokaryotic genome editing, gene regulation, and transcription recording, to which the other CRISPR-Cas systems may not be functional (Zebec et al., 2014; Peng et al., 2015; Li et al., 2016; Liu et al., 2018; Schmidt et al., 2018).

While type I, type II, and type V systems target DNAs (Barrangou et al., 2007; Brouns et al., 2008; Mulepati and Bailey, 2013; Anders et al., 2014; Jiang et al., 2015; Redding et al., 2015), and type VI systems target single-stranded RNAs (ssRNAs; East-Seletsky et al., 2016; Liu et al., 2017b,c; Shmakov et al., 2017), type III systems have been shown to have both DNA and RNA cleavage abilities both *in vivo* and *in vitro* (Marraffini and Sontheimer, 2008; Hale et al., 2009; Staals et al., 2014; Tamulaitis et al., 2014; Peng et al., 2015; Elmore et al., 2016; Ichikawa et al., 2017; Liu et al., 2017a; Tamulaitis et al., 2017). DNA target interference by type III systems requires the directional transcription of the target, as the DNase activity of the crRNPs is stimulated by base pairing between the guiding crRNAs and the transcript of the target DNAs (Deng et al., 2013; Goldberg et al., 2014; Samai et al., 2015; Elmore et al., 2016; Jiang et al., 2016; Liu et al., 2017a). Additionally, the Palm domain of Cas10 synthesizes cyclic oligoadenylates (cAns) as secondary messengers, which bind to CARF domain of Csx1 and activates the RNase activity of HEPN domain of Csx1 to non-specifically cleave the foreign DNA transcripts, and probably host transcripts as well (Kazlauskiene et al., 2017; Niewoehner et al., 2017; Foster et al., 2019). While PAM recognition is required for authentication of the interference process of type I and type II systems (Deveau et al., 2008; Mojica et al., 2009; Sternberg et al., 2014; Redding et al., 2015), target interference by type III systems tolerates a broad range of protospacer flanking sequences (Marraffini and Sontheimer, 2010; Elmore et al., 2016; Pyenson et al., 2017).

We accumulated breakthrough findings regarding adaptation of type III CRISPR-Cas systems in recent years. Here, We summarized our knowledge regarding CRISPR-Cas adaptation with the emphasis on type III systems, and discussed open questions for type III adaptation studies.

## Adaptation by Type I and Type II CRISPR-Cas Systems

CRISPR-Cas adaptation procedure includes protospacer selection, processing, and integration (Heler et al., 2014; Nussenzweig and Marraffini, 2020).

### Integration by Cas1–Cas2 Complex

A CRISPR array usually associates with *cas* genes, and each Cas protein participates in one or more major steps of the CRISPR-Cas system-mediated defense (Bhaya et al., 2011; Makarova et al., 2011). Cas1 and Cas2 proteins form a hexamer (four Cas1 monomers centered by two Cas2 monomers) both *in vivo* and *in vitro* (Nunez et al., 2014; Wright et al., 2017; Wan et al., 2019; Wilkinson et al., 2019), which is essential for adaptation of all tested CRISPR-Cas systems (Barrangou et al., 2007; Yosef et al., 2012; Nunez et al., 2014; Heler et al., 2015; Wei et al., 2015b; Fagerlund et al., 2017). Through the aid of this complex, the 3’-OH groups of the two strands of the prespacer (processed protospacer for integration) successively attack the junctions between the leader and the first repeat, and between the first repeat and the first pre-existing spacer (Nunez et al., 2015b; Rollie et al., 2015). By transesterification reactions, Cas1–Cas2 complex integrates the double-stranded prespacer into the CRISPR array, splitting the plus and the minus strand of the first repeat, and leaving two gaps (Nunez et al., 2015b; Rollie et al., 2015). DNA polymerase(s) and ligase(s) are thought to be required to fill the gap and finish the whole process. Since DNA polymerase I has been shown required for the type I adaptation in *Escherichia coli* (Ivancic-Bace et al., 2015), it is proposed to be the polymerase that fills the integration gap.

For several type I CRISPR-Cas systems, for example, the type I-E system in *E. coli* K12, Cas1 and Cas2 are the only two Cas proteins required for adaptation (Datsenko et al., 2012; Yosef et al., 2012; Diez-Villasenor et al., 2013; Nunez et al., 2014), while most type I systems and all studied type II systems require other Cas proteins for protospacer recognition or processing (Barrangou et al., 2007; Heler et al., 2015; Wei et al., 2015b; Liu et al., 2017d; Kieper et al., 2018; Lee et al., 2018, 2019; Shiimori et al., 2018; Almendros et al., 2019). Besides Cas proteins, the leader sequence and at least one repeat unit (Yosef et al., 2012; Wei et al., 2015a; Grainy et al., 2019; Kim et al., 2019), and integration host factor (IHF) and some other elements are also required to ensure the integration to happen at the correct position (Nunez et al., 2016; Wang et al., 2016; Fagerlund et al., 2017; Wright et al., 2017; Yoganand et al., 2017; Rollie et al., 2018).

### The Recognition, Selection, and Processing of the Proper Protospacers

For well-studied type I and type II CRISPR-Cas systems, the protospacers are selected along foreign DNAs by system-specific protospacer adjacent motifs (PAMs; Deveau et al., 2008; Mojica et al., 2009; Shah et al., 2013; Wang et al., 2015). PAM recognition is also required for the authentication of the interference process (Deveau et al., 2008; Mojica et al., 2009; Sternberg et al., 2014; Redding et al., 2015), by which the crRNP complexes of type I and type II systems can protect the CRISPR loci (containing the same sequence as the target) within its own genome from interference. The Cas1–Cas2 complex of the type I-E system in *E. coli* K12 is sufficient to recognize the ATG PAM upstream of protospacers (Datsenko et al., 2012; Yosef et al., 2012; Diez-Villasenor et al., 2013; Nunez et al., 2014); while some other type I systems require Cas4 to recognize PAM sequences (Kieper et al., 2018; Lee et al., 2018; Shiimori et al., 2018). Cas4 is a RecB-like nuclease (Zhang et al., 2012; Lemak et al., 2014), and has been shown to recognize PAM and determine the length and the orientation of the new spacers for some of the type I CRISPR-Cas systems (Kieper et al., 2018; Lee et al., 2018, 2019; Shiimori et al., 2018; Almendros et al., 2019; Zhang et al., 2019). The Cas9 protein of the type II system of *Streptococcus pyogenes* contains a PAM binding motif and performs PAM recognition to select the proper protospacers (Heler et al., 2015).

RecBCD complexes and their homologous protein complexes in prokaryotic cells bind to double-stranded DNA (dsDNA) breaks, and repair the broken DNAs by degradation and homologous recombination (Dillingham and Kowalczykowski, 2008). RecBCD complexes have been shown to be required for adaptation of some tested type I systems (Ivancic-Bace et al., 2015; Levy et al., 2015; Radovcic et al., 2018). Since dsDNA breaks frequently happen during DNA replication, extensively replicating invaders and the plasmids with high copy numbers become more sensitive than the cellular genome to adaptation (Ivancic-Bace et al., 2015; Levy et al., 2015). Moreover, RecBCD can be hampered by *chi* sequences (Dillingham and Kowalczykowski, 2008), and the enrichment of the *chi* sites around the replication termini of the prokaryotic genomes helps the adaptation machineries to more specifically recognize foreign DNAs (Ivancic-Bace et al., 2015; Levy et al., 2015).

The processing of the protospacers from the long substrates to the short and mature prespacers is a prerequisite of adaptation, but it is the least understood step of the adaptation process. The existence of 3′-single-stranded DNA (ssDNA) tails of the prespacers substantially facilitate adaptation (Arslan et al., 2014; Nunez et al., 2015a,b; Rollie et al., 2015, 2018; Van Orden et al., 2020). Cas1 is a non-specific exonuclease *in vitro* when associated with Cas2 in the adaptation complex (Wiedenheft et al., 2009; Babu et al., 2011; He et al., 2018; Radovcic et al., 2018), and it trims 5′ ends of the protospacers, leaving 3’-ssDNA tails for the following integration (Wang et al., 2015; Fagerlund et al., 2017). In *Streptococcus thermophilus*, Cas2 of the type I-E system possesses a DnaQ-like 3′-5′ exonuclease domain, which has been proposed to process the 3′-overhangs of the prespacers to promote integration (Drabavicius et al., 2018). Some other non-Cas exonucleases, including DnaQ and ExoT, have also been shown to be involved in the 3’-ssDNA tail generation of the prespacers (Ramachandran et al., 2020).

### Primed Adaptation

Target nucleic acids can escape from CRISPR-Cas-mediated interference by mutation(s) at pivotal positions within protospacers/targets or PAMs (Semenova et al., 2011; Westra et al., 2013). However, a pre-existing spacer in a CRISPR array, which is partially or totally complementary to a fragment of a molecule, can greatly stimulate adaptation against the same molecule (Datsenko et al., 2012; Swarts et al., 2012). To acquire new spacers from a molecule that the system has never processed before is termed “naïve adaptation,” whereas adaptation triggered by a pre-existing spacer (priming spacer) is termed “primed adaptation.” Primed adaptation is substantially more efficient than naïve adaptation (Fineran et al., 2014), and directs the adaptation machinery to the invader DNA instead of self-genome (Datsenko et al., 2012), thus providing the hosts with a co-evolutionary strategy to minimize the amount of CRISPR-Cas escapers. Primed adaptation has been studied and reported for many type I and two type II CRISPR-Cas systems, and interestingly, the secondarily adapted protospacers during primed adaptation were found to distribute around the cutting sites of crRNPs, with only one exception (type I-E system of *E. coli* K12; Datsenko et al., 2012; Swarts et al., 2012; Savitskaya et al., 2013; Fineran et al., 2014; Li et al., 2014; Richter et al., 2014; Nussenzweig et al., 2019; Garrett et al., 2020; Wiegand et al., 2020; Hoikkala et al., 2021; Mosterd and Moineau, 2021). The mechanism(s) of primed adaptation are still under research and debate.

## Adaptation by Type III CRISPR-Cas Systems

Many type III CRISPR-Cas systems, especially most type III-B systems, are not associated with *cas1* or *cas2* gene (Makarova et al., 2020a), so type III systems had been thought to be inert in adaptation for a long time. Instead, some type III systems appear to co-occur with type I systems and utilize crRNAs processed by the type I systems to provide additional defense against the invaders (Majumdar et al., 2015; Silas et al., 2017a). Until recent years, direct adaptation by type III systems has been observed and investigated.

### Reverse Transcriptase-Mediated Type III CRISPR-Cas Adaptation

**Figure 1:**
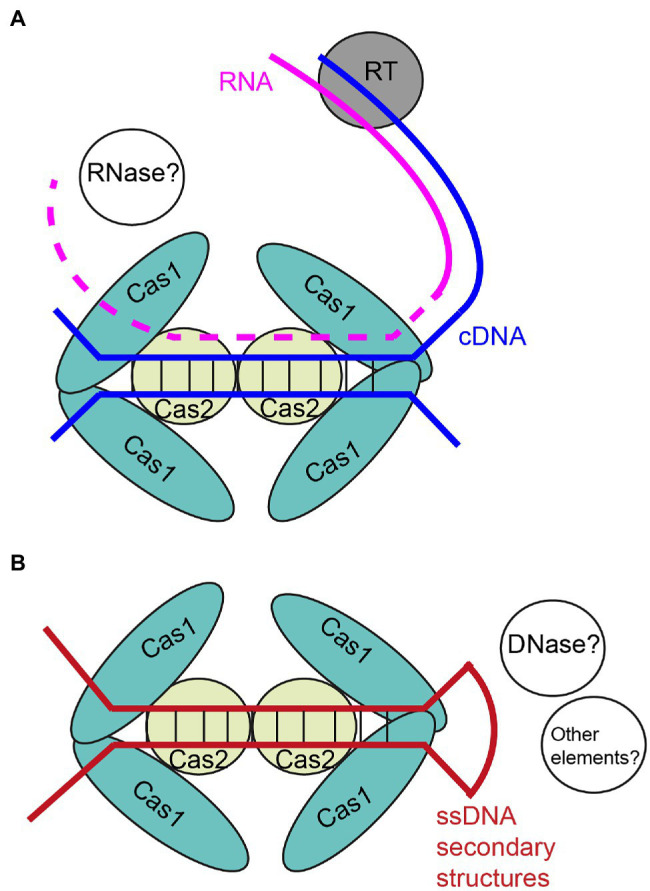
Diagrams of unique type III adaptation preference with **(A)** and without **(B)** reverse transcriptase activity. **(A)** RT represents reverse transcriptase, which is usually fused with Cas1–Cas2 complex, and may also be independent as well. Complementary DNA (cDNA) is depicted by blue. RNA template is depicted by pink, and the dashed lines represents potential digestion against the RNA template. **(B)** Single-stranded DNA (ssDNA) secondary structure is depicted by red. Hypothetical proteins or other elements are presented by white balls.

In 2016, Silas et al. (2016) reported adaptation by the type III-B system of *Marinomonas mediterranea*, revealing a novel reverse transcriptase (RT)-fused-Cas1 protein. While the reported RT-free systems can only adapt DNAs as CRISPR spacers, the type III-B system can use both RNAs and DNAs as substrates, and adaptation against RNAs is dependent on the RT (Figure 1A). This additional adaptation against RNAs makes the system preferentially acquire new spacers from highly transcribed regions versus weakly transcribed regions, which is beneficial for the function of the system, since target interference by type III systems requires transcription of the targets. Soon after this exciting finding, a similar RT-Cas1-Cas2 complex of *Fusicatenibacter saccharivorans* was used as a novel and efficient tool to record transcription event in *E. coli* (Schmidt et al., 2018). A similar RT-mediated type III adaptation against highly transcribed regions was reported by Gonzalez-Delgado et al. (2019), and moreover, they observed a dramatic preference against the coding strand of the rRNA genes. They speculated that the rRNA-encoding strand preference was also caused by RT and there was a correlation between gene transcription and new spacer orientation. However, since RT-active type III systems have no strand bias during adaptation against the other genes (Silas et al., 2016; Gonzalez-Delgado et al., 2019), it appears less likely that the bias was caused by transcription and RT activity. The findings by Zhang et al. (2021) and Aviram et al. (2022) indicate that the secondary structures formed by the coding strand of the rRNA genes (e.g., when the template strand is being processed by RNA polymerase) serve as additional and preferred substrates for CRISPR-Cas adaptation (see below).

### Type III CRISPR-Cas Adaptation Against Virulent Phages

The reported RT-encoding type III systems are not representative, because less than 10% of type III systems have RT activity (Silas et al., 2016). In 2020, Artamonova et al. (2020) observed and reported robust adaptation against a virulent phage, phiFa, by a RT-free type III system of *Thermus thermophilus*. The protospacers detected by high-throughput sequencing had a strand bias in that the template strands of the phage were adapted more extensively than the encoding strands, which was caused by counter-selection, since the crRNAs of the type III system needed to bind to the mRNAs of the phages to be functional. More interestingly, they found that the long terminal repeat (LTR), as the firstly invading part and early transcribed region of the phage, was adapted substantially more efficiently than the other parts of the phage, and the authors reasoned that maybe the LTR region encoded an anti-CRISPR element that blocked the functions of the CRISPR-Cas system. While not inconsistent with the data, it is more likely that the LTR formed secondary structures since it was a repeat-rich region, including palindromic, direct, and inverted repeats, and such structures could be recognized by type III CRISPR-Cas system (see below); or only adaptation against the early transcribed genes could perform timely defense against the phage. Soon after, Zhang et al. (2021) observed type III adaptation, followed by new crRNA-mediated defense, against virulent phage in *S. thermophilus* as well.

### Common and Unique Properties of Type III CRISPR-Cas Adaptation

In 2021, Zhang et al. (2021) for the first time provided a detailed analysis of the properties of type III CRISPR-Cas adaptation in *S. thermophilus*. The authors compared the patterns of adaptation by the type III-A and a type II-A CRISPR-Cas systems of *S. thermophilus* against different rolling circle replicating (RCR) plasmids and theta-replicating plasmids, as well as host genome. A prominent and unique feature of the adaptation by the type III system was the apparent recognition of the single-strand origins (*sso*s) of the RCR plasmids, contrasting with that of the type II system. RCR plasmids produce ssDNA intermediates during their replication, and the long and partially palindromic *sso*s form stem-loop structures to trigger the synthesis of the minus strand (Khan, 1997; Del Solar et al., 1998; Khan, 2000; Ruiz-Maso et al., 2015). The authors reasoned that the ssDNA hairpins served as additional and preferred dsDNA substrates for adaptation of the type III system (Figure 1B). Similarly, the partially palindromic *oriT* sequence of pNT1 plasmid, and the stem-loop structures enriched regulatory regions of the genomic and plasmid genes, as well as the cloverleaf structures enriched rRNA and tRNA encoding regions of self-genome, were also enriched in type III adaptation but not in type II adaptation (Zhang et al., 2021). Most of natural plasmids of gram-positive bacteria and many of those of gram-negative bacteria are RCR plasmids (Khan, 1997). Moreover, the crucial structure of *oriT* and other DNA secondary structures are important for the conjugative transfer and other functions of environmental mobile genetic elements (Bikard et al., 2010). As a consequence, secondary structure recognition by the type III CRISPR-Cas system can be beneficial for the system to specifically and efficiently eliminate the invaders. In 2022, Aviram et al. (2022) systematically studied the adaptation by a RT-free type III system of *Staphylococcus epidermidis* (expressed in *Staphylococcus aureus*). They observed similar adaptation preference against rRNA and tRNA encoding regions in host genome by the type III system, but not by the type II system in the same host, further supporting the reality of the unique property of type III adaptation.

There is no known reverse transcriptase encoding sequence in *S. thermophilus* genome, and Zhang et al. (2021) did not observe direct correlation between type III adaptation and DNA transcription in *S. thermophilus*. However, the authors did observe slight preference of type III adaptation against highly transcribed regions of plasmids, and constant preference against riboswitch transcriptional attenuators. These riboswitches lie in the 5’ UTR of the regulated mRNAs, and interaction between a signaling molecule and a riboswitch controls formation of a transcriptional terminator hairpin (Henkin, 2008). Besides, a general enrichment of type III spacers was observed roughly at 10–50 bp downstream from start codons of genomic genes. Moreover, for all the regions mentioned here, type III spacers were specifically enriched at the encoding strand, which was displaced as ssDNA when the template strand was occupied by transcription machinery. These findings further indicate that DNA secondary structures formed by ssDNAs can serve as additional and preferred substrates for type III adaptation (Figure 1B). While the type III-A system of *S. thermophilus* has no direct or obvious correlation between the adaptation and DNA transcription level, in contrast, the frequency of adaptation by type III-A system of *S. epidermidis* was found to be directly and obviously correlated with DNA transcription level (Aviram et al., 2022), in a similar way with the RT-active type III systems (Silas et al., 2016; Gonzalez-Delgado et al., 2019). It is possible that there is an unknown and intrinsic mechanism of the *S. epidermidis* type III-A system to target highly transcribed region during adaptation. In contrast, it is also possible that *S. aureus* cells potentially express unknown reverse transcriptase, after all, many *Staphylococcus* species are proposed to have putative reverse transcriptase encoding sequences, for examples, see NCBI accession CAC8888864.1 and UniProtKB D2J8E1. For some RT-active type III systems, RT domain is fused with Cas6 which is not related to adaptation, instead of Cas1 or Cas2 (Silas et al., 2016), implicating that RT domains do not have to be in the adaptation complexes to influence adaptation pattern; in contrast, independent cellular RTs may be able to generate additional substrates for type III adaptation as well (Figure 1A).

Like many investigated type I and type II systems, adaptation by the type III-A system of *S. epidermidis* was facilitated by DNA free ends, which was enhanced by AddAB DNA repairing complex (homologous to RecBCD) and hampered by *chi* sites (Aviram et al., 2022), indicating that this is a common feature for all or most CRISPR-Cas systems. The lengths of all tested type III spacers fell into a roughly normal distribution, centered by 36 bp (Silas et al., 2016; Gonzalez-Delgado et al., 2019; Artamonova et al., 2020; Zhang et al., 2021; Aviram et al., 2022). Direct adaptation by all tested type III systems are PAM-independent (Silas et al., 2016; Gonzalez-Delgado et al., 2019; Artamonova et al., 2020; Zhang et al., 2021; Aviram et al., 2022), and requires only Cas1 and Cas2 proteins, but not Cas6 or any interference-related Cas proteins (Silas et al., 2016; Schmidt et al., 2018; Zhang et al., 2021; Aviram et al., 2022).

**Figure 2:**
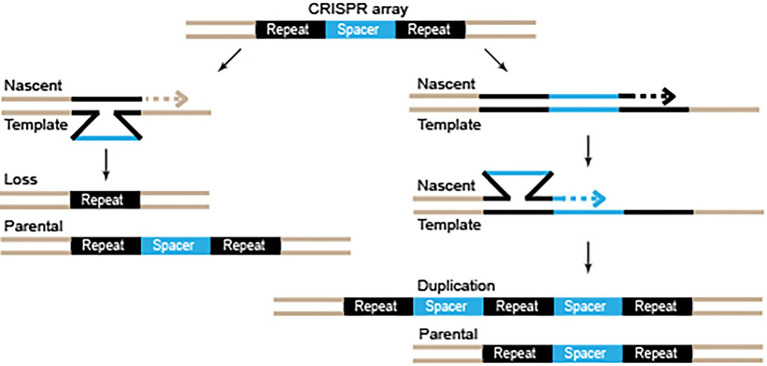
Diagram of repeat-spacer loss and duplication by DNA replication slippage. Repeats and spacers are depicted by black and blue. Dashed arrows indicate the DNA synthesis direction.

Intriguingly, although adaptation was inert after knocking out *cas1* or *cas2* genes, Zhang et al. (2021) observed the duplication of the repeat and the pre-existing spacer units, revealing an adaptation-independent repeat-spacer replication event. Such replication was observed in both the type III and the type II systems of *S. thermophilus*, indicating that it is a universal feature of all or many of the CRISPR-Cas systems (Zhang et al., 2021). DNA replication slippage in the repeat-rich region may help the CRISPR-Cas systems to replicate recently acquired spacers to enhance the expression of the crRNAs, as well as to lose the old spacers to keep a compact CRISPR array (Figure 2). While the analyses in the research by Zhang et al. (2021) were unable to detect spacer loss, such loss had been observed in a study regarding a type I CRISPR-Cas system (Rao et al., 2017).

## Conclusion and Open Questions

As a conclusion, different labs have independently detected adaptation against plasmids, phages, and host genomes by type III CRISPR-Cas system (Silas et al., 2016, 2017b; Schmidt et al., 2018; Gonzalez-Delgado et al., 2019; Artamonova et al., 2020; Zhang et al., 2021; Aviram et al., 2022). Interesting common and unique properties of type III adaptation have been identified. However, there are still interesting and important questions unanswered regarding type III adaptation. (1) We do not fully understand the detailed procedure or entire mechanism of RT-mediated type III adaptation. Does RT-mediated adaptation happen during or after the RT reaction? How do those adaptation modules process the DNAs after RT reaction? Is it necessary for the cells to digest the template RNA before CRISPR-Cas adaptation (Figure 1A)? These questions remain to be answered. (2) Whether type III Cas1–Cas2 complex has the intrinsic ability to recognize secondary structures, or other non-Cas elements are involved in this recognition, remains to be studied. (3) The mechanism of adaptation-independent dynamics of CRISPR arrays and the benefits of the process remain to be studied. (4) Whether primed adaptation activity exists in type III systems, and the mechanism of type III primed adaptation, remain to be studied. Target interference of type III systems tolerates a broad range of PAMs (Marraffini and Sontheimer, 2010; Elmore et al., 2016; Pyenson et al., 2017), and also tolerates apparently more mutations within the targets than type I and type II systems (Maniv et al., 2016; Pyenson et al., 2017). As a result, type III systems minimize the potential escapers of the invading nucleic acids (Maniv et al., 2016; Pyenson et al., 2017). Despite this difficulty of escape, primed adaptation may still be beneficial for type III CRISPR-Cas-mediated defense. As discussed above, naïve adaptation by the type III system preferentially uptakes the protospacers at the encoding strands of the promoter regions of expressed genes (Zhang et al., 2021). Since the target interference ability of the type III system requires a reverse complementary RNA, DNA uptake against the encoding strand will not directly contribute to defense. Moreover, as to the *bona fide* protospacers derived from the template strands, if the protospacer region was weakly transcribed or a late transcript in phage infection, the type III spacer-mediated defense may be insufficient to efficiently clear phage or plasmid nucleic acids (Goldberg et al., 2014; Rostol and Marraffini, 2019). In these situations, the potential primed adaptation triggered by the “inefficient” spacers may be able to provide a chance to the system to perform efficient secondary uptake to counter against the invaders.

## Author Contributions

XZ revised and wrote this review. XA revised this review. All authors contributed to the article and approved the submitted version.

## Funding

This work was supported by the China National Key R&D Program during the 14th Five year Plan Period (2021YFD2200101), the National Natural Science Foundation of China (31870652, 31570661).

## Conflict of Interest

The authors declare that the research was conducted in the absence of any commercial or financial relationships that could be construed as a potential conflict of interest.

## Publisher’s Note

All claims expressed in this article are solely those of the authors and do not necessarily represent those of their affiliated organizations, or those of the publisher, the editors and the reviewers. Any product that may be evaluated in this article, or claim that may be made by its manufacturer, is not guaranteed or endorsed by the publisher.
